# Social Media Detox and Youth Mental Health

**DOI:** 10.1001/jamanetworkopen.2025.45245

**Published:** 2025-11-24

**Authors:** Elombe Calvert, Maddalena Cipriani, Bridget Dwyer, Victoria Lisowski, Jane Mikkelson, Kelly Chen, Matthew Flathers, Christine Hau, Winna Xia, Juan Castillo, Alex Dhima, Sean Ryan, John Torous

**Affiliations:** 1 Department of Psychiatry, Beth Israel Deaconess Medical Center, Boston, Massachusetts; 2 Department of Psychology, University of Bath, Bath, United Kingdom

## Abstract

**Importance:**

The association between social media use and youth mental health remains poorly understood, with recent systematic reviews reporting inconsistent and conflicting findings. These discrepancies reflect the overreliance on self-reported estimates of use, lack of passive monitoring of behavior, and limited measurement of momentary mental health states.

**Objectives:**

To examine the association between objective social media use, problematic engagement, and mental health outcomes in young adults, as well as evaluate the outcomes of a 1-week social media detox intervention on behavior and mental health symptoms.

**Design, Setting, and Participants:**

A remote cohort study conducted in the US using a national recruitment registry between March 2024 and March 2025, where participants completed a 2-week observational baseline, followed by an optional 1-week social media detox intervention. Participants were young adults (aged 18-24 years) with a smartphone and English fluency.

**Exposures:**

Social media use of Facebook, Instagram, Snapchat, TikTok, and X over a 2-week baseline period, followed by an optional 1-week detox intervention.

**Main Outcomes and Measures:**

The main outcomes were detox changes in symptoms of depression (Patient Health Questionnaire-9), anxiety (Generalized Anxiety Disorder-7), insomnia (Insomnia Severity Index), and loneliness (University of California, Los Angeles Loneliness Scale), as well as within-participant changes in behavior including screen use, communication, mobility, and momentary mental health states.

**Results:**

Of 417 enrolled participants, 373 (mean [SD] age, 21.0 [1.9] years) completed baseline assessments, with 295 (79.1%) opting into a detox intervention that reduced symptoms anxiety by 16.1% (−1.9 reduction; Cohen *d*, −0.44; 95% CI, −0.56 to −0.32), depression by 24.8% (−2.0 reduction; Cohen *d*, −0.37; 95% CI, −0.49 to −0.32) and insomnia by 14.5% (−2.1 reduction; Cohen *d*, −0.44; 95% CI, −0.56 to −0.32). There was no significant change in loneliness (Cohen *d*, −0.40; 95% CI, −0.17 to 0.06). Marginal increases were seen in home time (β, 42.8; 95% CI, 24.3 to 61.2 minutes) and screen duration (β, 15.4; 95% CI, 4.9 to 25.9 seconds), with considerable within-person variability. No other changes in behavioral or EMA-based features were observed after the detox.

**Conclusions and Relevance:**

In this cohort of young adults, reducing social media use for 1 week was associated with reductions in symptoms of depression, anxiety, and insomnia; however, the durability of these therapeutic outcomes and their associations with behavior warrant further study, particularly in more diverse populations.

## Key Points

**Question:**

Is reducing social media use associated with behavior and mental health outcomes among young adults?

**Findings:**

In this cohort study of 373 participants, problematic use of social media was found to be significantly associated with worse mental health outcomes. A 1-week social media detox intervention significantly reduced symptoms of anxiety by 16.1%, depression by 24.8%, and insomnia by 14.5%. Although statistically detectable, increases in home time and screen duration were small compared with large within-person variabilities in behavior.

**Meaning:**

These findings suggest that reducing social media use for 1 week may improve mental health outcomes in young adults; however, the durability of these therapeutic outcomes and their associations with behavior require further investigation.

## Teaser

This cohort study evaluates the outcomes of a 1-week social media detox intervention on behavior and mental health symptoms.

## Introduction

The association between social media use and youth mental health is complex and remains poorly understood. Recent systematic reviews and meta-analyses report inconsistent and conflicting associations.^1,2,3,4,5,6^ While these discrepancies may partly reflect the differences in how young people engage with social media,^7^ they are also attributable to the field’s overreliance on only self-reported estimates of use (eg, screen time^8^) and related behaviors (eg, communication habits,^9^ sleep patterns,^10^ and physical activity^11^), which introduce bias and confound associations.^12^

Given that self-reported screen time measures have demonstrated weak associations with youth mental health,^5^ there is growing recognition of the need for more precise measurement approaches (eg, compulsivity and emotional dependence). Objective data, when complemented by assessments that capture the psychological and behavioral dimensions of problematic use,^13^ although largely self-reported, may provide a more comprehensive understanding of how social media affects youth mental health.

Equally important is the need to examine behaviors associated with social media use.^14^ Passive behavioral features derived from smartphone sensors (eg, mobility patterns, communication, sleep, and screen use) have shown associations with mental health symptoms^15^ and can complement traditional assessments by capturing aspects of functioning not easily measured by self reports. By capturing changes in behavior as they occur, they complement retrospective self reports and provide ecologically valid insights into the daily experiences of young adults. Together, these approaches offer a more comprehensive and rigorous framework for advancing our understanding of social media’s association with youth mental health.

Digital phenotyping, defined as the moment-by-moment quantification of the individual-level human phenotype in situ using data from personal digital devices,^16^ provides a framework for combining objective social media use data, passively collected behavioral signals, and in-the-moment self-reports of mental health via ecological momentary assessment (EMA). This multimodal approach enables a more comprehensive characterization of youth social media engagement and directly addresses methodological concerns raised in recent meta-analytic findings.^12^ Given that smartphones are the primary medium through which young people access social media, and digital phenotyping can operate passively and unobtrusively on these devices, it offers a scalable and low-cost solution for capturing social media use in clinical practice settings.^16^ Although passive sensing has been applied in observational research,^17^ there has been less attention toward its potential to examine behaviors associated with objective and problematic social media use.

Young adults (aged 18-24 years) have been consistently identified in the literature as among the most frequent users of social media^18^ and as a group at heightened risk for the onset of depression, anxiety, and related mental health concerns.^19,20^ This developmental stage, often described as emerging adulthood, is marked by heightened vulnerability and transition, making it a particularly important population for examining the outcomes of social media use. Short-term interventions, such as a 1-week social media detox, have been increasingly examined as feasible strategies for reducing problematic use and improving well-being in young populations, with several studies demonstrating measurable effects within this timeframe.^21,22,23^ Thus, we implemented a design that included a 2-week observational period, sufficient to establish baseline behavioral patterns^24^ followed by a 1-week detox intervention, to assess the association between reduced social media use, behavior, and mental health.

The present study integrates smartphone-based digital phenotyping, validated self-reports of problematic social media use, EMAs of in-the-moment symptoms, and standardized mental health surveys. We aimed to (1) compare the strength of associations between objective and self-reported measures of problematic social media use and mental health; (2) evaluate changes in symptoms of depression, anxiety, loneliness, and insomnia following a 1-week detox intervention; and (3) assess whether reductions in social media use were associated with changes in behavior and momentary mental health states.

## Methods

### Participants and Study Design

This study used a prospective cohort design with a voluntary intervention to evaluate the outcomes of a 1-week social media detox on the mental health of young adults aged 18 to 24 years. Participants were recruited through ResearchMatch (eMethods in Supplement 1). Eligibility criteria included ownership of an Android or iOS smartphone compatible with the mindLAMP application,^19^ ability to participate in virtual study visits, communicate in English, and provide informed consent. The study involved a 2-week observational phase with passive smartphone sensing, daily EMAs, and weekly standardized clinical surveys, followed by an optional 1-week social media detox intervention targeting 5 platforms (Facebook, Instagram, Snapchat, TikTok, and X [formerly Twitter]). Digital phenotyping and survey data were collected using the application and REDCap. Participants reported app-level usage metrics through their device settings. Participants were compensated with up to $150 US, prorated on the proportion of surveys completed. The study was approved by the Beth Israel Deaconess Medical Center review board and adhered to the Strengthening the Reporting of Observational Studies in Epidemiology (STROBE) reporting guideline for cohort studies.

### Study Procedures

Participants completed 3 virtual visits (eFigure 1 in Supplement 1). At visit 1, participants provided electronic informed consent and completed the first battery of baseline demographic, clinical, and problematic social media use assessments and configured the study’s digital phenotyping app (eFigure 2 in Supplement 1) on their smartphones. During the 2-week observational phase, participants completed daily EMAs while passive smartphone sensing data (eg, GPS, accelerometer, and screenstate) were continuously collected. At visit 2, participants repeated the same battery of assessments (excluding demographics) from visit 1, reviewed their personalized data summary with a research assistant (eFigure 3 in Supplement 1), and reported their app-level usage metrics for the previous 2 weeks by navigating to their device settings (eFigure 4, eFigure 5 in Supplement 1). Participants then self-selected into a 1-week social media detox intervention, continuing passive sensing and daily EMA surveys. At visit 3, participants repeated the same assessments as visit 2, reviewed their final data report, and documented their app-level usage for the detox week. To promote data integrity, participants were instructed during onboarding and at follow-up visits not to reset their phone usage counters and to self-report social media use across the 5 apps on alternate devices. A safety protocol was in place to escalate any identified clinical risk (eg, Patient Health Questionnaire-9 [PHQ-9] item 9 ≥ 2).

### Study Measures

Race and ethnicity were self-reported by participants at baseline on the enrollment survey. Response options for race included American Indian or Indigenous, Asian or Asian American, Black or African American, Middle Eastern or North African, Pacific Islander or Native Hawaiian, White or European American, multiracial or mixed race, and other. Ethnicity was categorized separately as Hispanic or not Hispanic. Age, gender, race, and ethnicity were assessed to account for potential confounding by social and demographic factors.

The median (IQR) proportion of passive sensing features that were nonmissing per participant was 0.59 (0.22–0.71), with a GPS-based passive data quality of 0.78 (0.32-0.96). Passive sensing features included measures of mobility (home time, entropy, and step count), screen interaction (screen duration, screen wakes, screen unlocks), and the frequency and diversity in communication (incoming text number, outgoing text number, text degree [the number of people texted], incoming call number, outgoing call number, call duration, and call degree [the number of different people called]). Mobility measures are feature-engineered from GPS and accelerometer data using the open-source Python library, Cortex.^25^ The median (IQR) daily EMA completion rate was 0.76 (0.67–0.80), with 351 of 373 participants (94%) responding to more than half of their EMA prompts. EMA measures captured daily mood, anxiety, and difficulty functioning. Brief descriptions of each feature and its derivation are provided in eMethods in Supplement 1.

Standardized surveys included the PHQ-9,^26^ Generalized Anxiety Disorder-7 (GAD-7),^27^ Insomnia Severity Index (ISI),^28^ UCLA Loneliness Scale (University of California Los Angeles-LS),^29^ Problematic Use of Social Networks Scale (PUSNS),^30^ Bergen Social Media Addiction Scale (BSMAS),^31^ Negative Social Media Comparison Scale (NSMCS),^32^ and Rosenberg Self-Esteem Scale.^33^ Objective social media features included platform-specific metrics such as mean daily screen time, pickups, notifications, and the number of days apps were opened. Details of scales and questions are provided in eMethods in Supplement 1.

### Statistical Analysis

We computed descriptive statistics to summarize baseline demographic characteristics, clinical measures, and social media use variables. Differences between participants who engaged in the detox vs no detox groups were examined using χ^2^ or Fisher exact tests for categorical variables and Mann-Whitney U tests for continuous variables.

Bivariate correlations (Pearson *r*) were calculated to examine associations between objective social media use, self-reported problematic social media use, and mental health outcomes. We then fit multivariable linear regression models for each outcome measure (PHQ-9, GAD-7, ISI, and UCLA-LS) to assess the independent contributions of objective and self-reported problematic social media use, adjusting for age, gender, education, race and ethnicity, and phone operating system.

Changes in clinical symptoms before and after the detox intervention were assessed using paired *t* tests, and Cohen d^34^ was calculated to estimate effect sizes. Sensitivity analyses were conducted by baseline depression symptom severity to examine potential differential outcomes of the detox intervention.

To estimate behavioral changes in digital phenotyping and EMA features, we fit 17 separate linear mixed-effects models (one per outcome), each including a random intercept to account for within-participant correlation across repeated daily observations. For each outcome feature Y*_it_* measured for participant *i* on day *t:*

*Y_it_* = + β_1 _Period*_it_* + β_2 _Age*_i_* + β_3 _Gender*_i_* + β_4 _Education*_i_* + β_4 _Education*_i_* + β_5 _Race*_i_* + β_6 _PhoneType*_i_* + *u_0i_* + ε*_it_*

where β_0_ is the fixed intercept, *u_0i_* is a participant-specific random intercept, and *ε_it_* is the residual error. Period (baseline vs detox week) was included as the main fixed effect, with covariates for age, gender, education, race, and phone type. This enabled the estimation of the mean detox effect across participants while accommodating for individual baseline variability. Given that 17 mixed-effects models were made, *P* values were corrected for making multiple comparisons using the Benjamini-Hochberg procedure. To understand how participants’ geographic location affected our estimates of detox behavior change, sensitivity analyses accounting for spatial confounding were completed (eMethods in Supplement 1).

Missingness in digital phenotyping features was assumed to be missing at random based on the Hawkins test.^35^ Missing data were addressed using multiple imputation by chained equations (MICE),^36^ generating 5 imputed datasets for statistical modeling. All *P* values were from 2-sided tests, with statistical significance determined at a false discovery rate-adjusted threshold of *P* < .05 using the Benjamini-Hochberg procedure. Statistical analyses were conducted using R version 4.4.1 in RStudio version 2024.04.2 (R Project for Statistical Computing).^37^

## Results

### Baseline Characteristics

A total of 417 participants enrolled in the study (eFigure 6 in Supplement 1), with 19 (4.6%) disqualified (fraudulent or non-US addresses), yielding an eligible sample of 398 participants. Of the 398, 25 (6.3%) withdrew, resulting in an analytic sample of 373 participants (Table 1). The cohort was predominantly female (277 participants [74.3%]), with a mean (SD) age of 21 (2) years. Most participants were undergraduate students (287 participants [76.9%]), followed by postgraduates (54 participants [14.5%]) and those with only a high school education (32 participants [8.6%]). The racial composition was as follows: 87 (23.3%) Asian or Asian American; 22 (5.9%) Black or African American; 29 (7.8%) Hispanic or Latinx; 8 (2.1%) Middle Eastern or North African; 26 (7.0%) multiracial or mixed race; 1 (0.3%) Pacific Islander or Native Hawaiian; 198 (53.1%) White; and 2 (0.5%) other; 337 (90.3%) owned an iOS smartphone. Baseline assessments were completed by 373 of 398 eligible participants (93.7%), with 295 (79.1%) opting into the 1-week detox intervention and 78 (20.9%) opting out (eFigure 6 in Supplement 1). Those who opted out of the detox did not differ significantly from those who participated in terms of demographic characteristics, baseline clinical measures (Table 1), social media screen time (eFigure 8 in Supplement 1), or problematic social media use scores. At baseline, all participants reported minimal depression (median [IQR] PHQ-9 score, 4.0 [2.0-8.0]) and anxiety (median [IQR] GAD-7 score, 4.0 [2.0-8.0]), no clinically significant insomnia (median [IQR] ISI score, 6.0 [3.0-10.0]), and moderate loneliness (median [IQR] UCLA-LS score, 36.0 [29.0-44.0]). See eTable 1 in Supplement 1 for comprehensive report of baseline measures.

**Table 1.** Participant Baseline Characteristics by Detox Status

Characteristic	Participants, No. (%)			*P* value^a^
	Total (N = 373)	Detox (n = 295)	No detox (n = 78)	
Age, mean (SD), y	21 (2)	22 (2)	21 (2)	.11
Gender^b^				
Female	277 (74.3)	220 (74.6)	57 (73.1)	.60
Male	73 (19.6)	59 (20.0)	14 (17.9)	
Nonbinary	12 (3.2)	8 (2.7)	4 (5.1)	
Transgender	9 (2.4)	6 (2.0)	3 (3.9)	
Other	2 (0.5)	2 (0.7)	0	
Education^c^				
College senior	146 (39.1)	125 (42.4)	21 (26.9)	.10
Postgraduate	54 (14.5)	41 (13.9)	13 (16.7)	
College sophomore	53 (14.2)	41 (13.9)	12 (15.4)	
College junior	51 (13.7)	41 (13.9)	10 (12.8)	
College freshman	37 (9.9)	23 (7.8)	14 (17.9)	
High school graduate	32 (8.6)	24 (8.1)	8 (10.3)	
Race and ethnicity^b^				
Asian or Asian American	87 (23.3)	74 (25.1)	13 (16.7)	.60
Black or African American	22 (5.9)	16 (5.4)	6 (7.7)	
Hispanic or Latinx	29 (7.8)	23 (7.8)	6 (7.7)	
Middle Eastern or North African	8 (2.1)	7 (2.4)	1 (1.3)	
Pacific Islander or Native Hawaiian	1 (0.3)	1 (0.3)	0	
White	198 (53.1)	152 (51.5)	46 (59.0)	
Multiracial or mixed race	26 (7.0)	21 (7.1)	5 (6.4)	
Other	2 (0.5)	1 (0.3)	1 (1.3)	
Phone type^c^				
iOS	337 (90.3)	264 (89.5)	73 (93.6)	.10
Android	36 (9.7)	31 (10.5)	5 (6.4)	
Clinical scores, median (IQR)^d^				
General Anxiety Disorder-7	4.0 (2.0-8.0)	4.0 (2.5-9.0)	4.0 (2.5-8.0)	>.90
Patient Health Questionnaire-9	4.0 (2.0-8.0)	4.0 (2.0-9.0)	4.0 (2.5-8.0)	.60
Insomnia Severity Index	6.0 (3.0-10.0)	7.0 (3.5-11.0)	6.0 (3.0-10.0)	.40
UCLA Loneliness Scale	36.0 (29.0-44.0)	36.0 (29.0-45.0)	38.0 (29.5-45.0)	.80

Abbreviation: UCLA, University of California, Los Angeles.

^a^ Significance tested at α < .05 from 2-tailed tests.

^b^ Fisher exact test.

^c^ χ^2^ Test.

^d^ Mann-Whitney *U* test.

### Baseline Social Media Use

During the 2-week baseline period, 350 participants (93.8%) used at least 1 of the 5 social media platforms included in the study, with only 23 participants (6.2%) reporting not using any. Participants had a mean (SD) screen time of 1.9 (1.4) hours per day across all 5 social media platforms (eFigure 7 in Supplement 1), corresponding to a mean (SD) of 26.5 (20.7) hours of cumulative use per participant over the 2-week period. Mean (SD) daily screen time was higher in female participants (2.0 [1.5] hours) than in male participants (1.3 [1.2] hours) (*P* < .001), with no other significant differences observed by race, educational status, phone operating system, or detox opt-in status (eFigure 8 in Supplement 1).

### Detox Social Media Use

The 1-week social media detox intervention was completed by 295 participants (79.1%). During the intervention period, mean (SD) daily social media screen time decreased to 0.5 (0.8) hours, a significant reduction from baseline use of 1.9 (1.4) hours (*P* < .001). Most participants (265 of participants [89.8%]) reduced their screen time during the detox, with a mean (SD) total reduction of 9.2 (9.2) hours per participant (Figure). Nonadherence rates varied by platform, with Instagram (200 of 295 participants [67.8%]) and Snapchat (144 participants [48.8%]) showing the highest rates of continued use during the detox period (eFigure 9 in Supplement 1). Participants were more likely to reduce their use of TikTok (188 participants [63.7%]) than Instagram (95 participants [32.2%]) or Snapchat (151 participants [51.2%]). X and Facebook had the highest adherence rates, with 243 (82.4%) and 216 (73.2%) participants, respectively, reducing their screen time.

**Figure.**
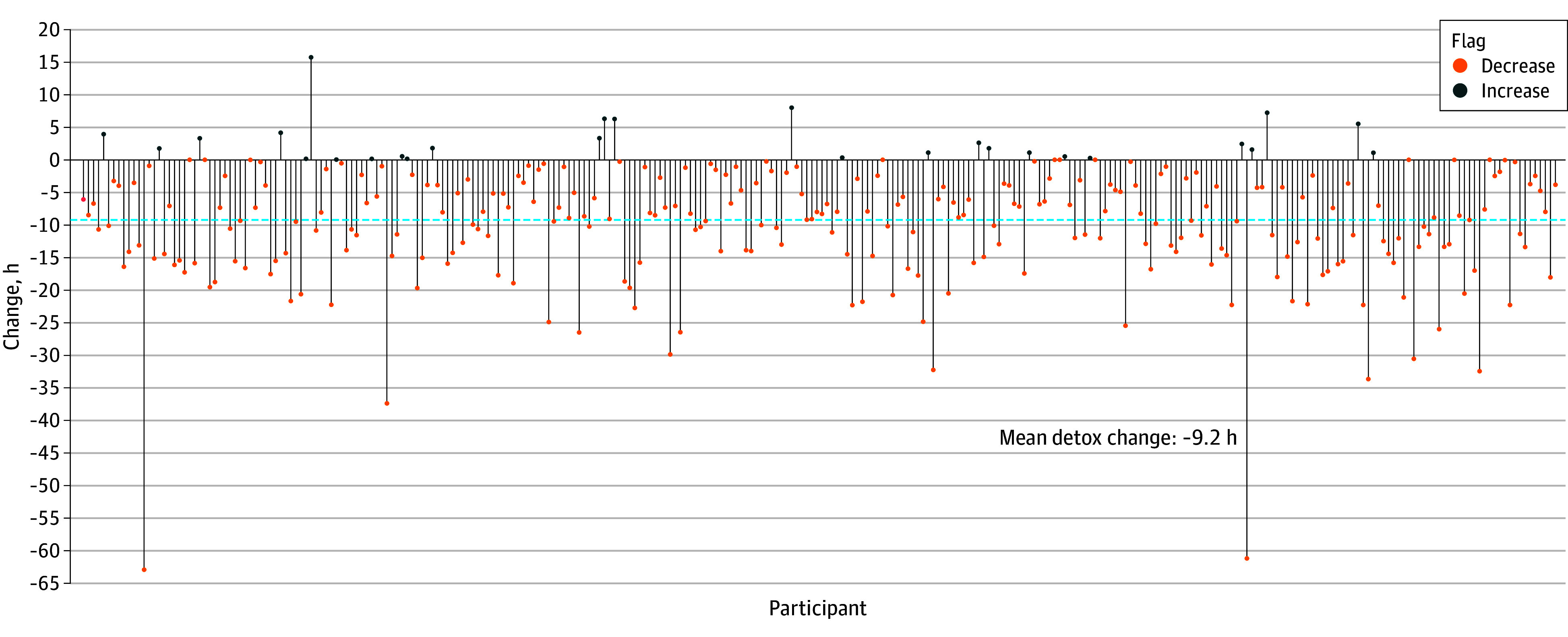
Participant-Level Change in Mean Daily Social Media Screen Time During Detox Each vertical line represents the change in screen time (hours) per participant from baseline to postdetox period. The blue dashed line highlights the mean change of −9.2 hours, demonstrating an overall reduction in social media use during detox.

### Social Media Use and Mental Health

Overall, small positive correlations were observed between objective social media use and self-reported problematic social media use, with correlation coefficients ranging from *r* = 0.14 to *r* = 0.32 (eTable 2 in Supplement 1). Self-reported problematic social media use exhibited stronger correlations with mental health features than objective social media use (eTable 3 in Supplement 1). Significant associations were observed between problematic use (PUSNS) and PHQ-9 (*r* = 0.48; *P* < .001), PUSNS and GAD-7 (*r* = 0.41; *P* < .001), NSMCS and UCLA-LS (*r* = 0.49; *P* < .001), and addictive use (BSMAS) and ISI (*r* = 0.35; *P* < .001). In contrast, objective social media use showed only weak correlations with baseline mental health outcomes, including total daily screen time and ISI (*r* = 0.15; *P* = .02), and Instagram notifications with both ISI (*r* = 0.15; *P* = .03) and PHQ-9 (*r* = 0.15; *P* = .03).

Given the observed associations in bivariate analyses, multivariable linear regression models were constructed for each clinical outcome to evaluate these associations while adjusting for baseline covariates (Table 2). Across all adjusted models, self-reported PSMU was consistently associated with greater mental health symptom severity. Notably, higher BSMAS scores were associated with greater depressive symptoms (β = 0.29; 95% CI, 0.15 to 0.43; *P* < .001), higher NSMCS scores with greater anxiety (β = 0.20; 95% CI, 0.11 to 0.29; *P* < .001), higher PUSNS scores with greater loneliness (β = 0.21; 95% CI, 0.04 to 0.37; *P* = .01), and higher NSMCS scores with greater insomnia symptoms (β = 0.20; 95% CI, 0.04 to 0.37; *P* = .02). In contrast, objective social media use including screen time, pickups, notifications, and days opened, were not significantly associated with clinical outcomes, apart from a few small inverse associations, such as between screen time and anxiety (β = −0.01; 95% CI, −0.02 to –0.01; *P* < .001).

**Table 2.** Pooled Multivariable Regression Estimates of the Associations Between Self-Reported and Objective Social Media Use and Mental Health Outcomes

Variable	Model 1: PHQ-9^a^		Model 2: GAD-7^b^		Model 3: UCLA-LS^c^		Model 4: ISI^d^	
	β (95% CI)	*P* value^e^	β (95% CI)	*P* value^e^	β (95% CI)	*P* value^e^	β (95% CI)	*P* value^a^
Baseline characteristics								
Age	0.19 (−0.15 to 0.52)	.27	−0.10 (−0.45 to 0.24)	.56	0.52 (−0.29 to 1.33)	.21	0.07 (−0.34 to 0.47)	.75
Gender								
Female	1 [Reference]	NA	1 [Reference]	NA	1 [Reference]	NA	1 [Reference]	NA
Male	0.57 (−0.56 to 1.70)	.32	−0.74 (−1.97 to 0.49)	.24	2.59 (−0.09 to 5.27)	.06	1.50 (0.13 to 2.88)	.03
Nonbinary	5.24 (2.88 to 7.59)	<.001	2.21 (−0.24 to 4.66)	.08	9.97 (4.31 to 15.63)	<.001	6.18 (3.31 to 9.05)	<.001
Transgender	3.81 (1.05 to 6.56)	.01	1.96 (−0.90 to 4.83)	.18	10.29 (3.83 to 16.75)	.002	5.51 (2.16 to 8.87)	.001
Other	3.18 (−2.45 to 8.82)	.27	4.48 (−1.38 to 10.34)	.13	13.68 (0.49 to 26.87)	.04	1.08 (−5.79 to 7.95)	.76
Education								
College senior	1 [Reference]	NA	1 [Reference]	NA	1 [Reference]	NA	1 [Reference]	NA
Postgraduate	−0.24 (−1.53 to 1.04)	.71	0.48 (−0.92 to 1.88)	.50	1.50 (−1.78 to 4.77)	.37	0.27 (−1.30 to 1.85)	.73
College junior	0.14 (−1.31 to 1.58)	.85	−0.27 (−1.77 to 1.24)	.73	−1.15 (−4.70 to 2.40)	.52	−0.43 (−2.20 to 1.33)	.63
College sophomore	0.50 (−0.93 to 1.93)	.49	1.74 (0.25 to 3.23)	.02	2.33 (−1.38 to 6.04)	.22	−0.01 (−1.76 to 1.74)	.99
College freshman	0.30 (−1.63 to 2.22)	.76	0.08 (−1.92 to 2.08)	.94	−0.50 (−5.11 to 4.12)	.83	0.26 (−2.09 to 2.61)	.83
High school graduate	0.13 (−1.85 to 2.11)	.90	−0.85 (−2.91 to 1.21)	.42	0.93 (−3.94 to 5.80)	.71	−0.42 (−2.83 to 2.00)	.73
Race and ethnicity								
White	1 [Reference]	NA	1 [Reference]	NA	1 [Reference]	NA	1 [Reference]	NA
Asian or Asian American	0.39 (−0.66 to 1.43)	.47	0.22 (−0.87 to 1.30)	.69	2.07 (−0.38 to 4.51)	.10	1.66 (0.38 to 2.93)	.01
Hispanic or Latinx	0.73 (−0.95 to 2.41)	.39	0.88 (−0.87 to 2.63)	.32	4.64 (0.69 to 8.59)	.02	0.86 (−1.20 to 2.91)	.41
Black or African American	2.59 (0.69 to 4.50)	.01	1.35 (−0.64 to 3.33)	.18	5.27 (0.78 to 9.77)	.02	2.70 (0.38 to 5.02)	.02
Middle Eastern or North African	0.84 (−2.04 to 3.71)	.57	−0.07 (−3.83 to 3.69)	.97	7.78 (−3.32 to 18.88)	.15	5.71 (2.04 to 9.38)	.003
Pacific Islander or Native Hawaiian	2.90 (−4.96 to 10.75)	.47	2.25 (−5.92 to 10.43)	.59	−0.29 (−18.69 to 18.12)	.98	4.56 (−5.01 to 14.14)	.35
Multiracial or mixed race	1.51 (−0.16 to 3.18)	.08	−0.53 (−2.26 to 1.20)	.55	2.72 (−1.27 to 6.72)	.18	0.15 (−1.88 to 2.18)	.89
Other	1.25 (−4.31 to 6.82)	.66	0.42 (−5.37 to 6.21)	.89	−3.50 (−16.51 to 9.52)	.60	3.65 (−3.14 to 10.44)	.29
Phone type								
iOS	1 [Reference]	NA	1 [Reference]	NA	1 [Reference]	NA	1 [Reference]	NA
Android	−0.31 (−1.82 to 1.20)	.69	−0.86 (−2.44 to 0.72)	.28	4.44 (0.74 to 8.15)	.02	−0.79 (−2.63 to 1.05)	.40
Self-reported PSMU								
BSMAS	0.29 (0.15 to 0.43)	<.001	0.23 (0.08 to 0.37)	<.001	−0.03 (−0.36 to 0.30)	.87	0.20 (0.04 to 0.37)	.02
PUSNS	0.03 (−0.04 to 0.10)	.34	<0.01 (−0.07 to 0.08)	.89	0.21 (0.04 to 0.37)	.01	0.04 (−0.04 to 0.13)	.32
NSMCS	0.14 (0.06 to 0.23)	<.001	0.20 (0.11 to 0.29)	<.001	0.45 (0.25 to 0.65)	<.001	0.07 (−0.03 to 0.17)	.18
Objective SMU								
Aggregate TDS	−0.01 (−0.01 to 0.01)	.14	−0.01 (−0.02 to −0.01)	.001	−0.02 (−0.04 to −0.01)	.03	<0.01 (−0.01 to 0.01)	.64
Aggregate TDP	0.01 (−0.01 to 0.02)	.45	0.01 (−0.01 to 0.03)	.33	−0.01 (−0.06 to 0.04)	.77	<-0.01 (−0.02 to 0.02)	.89
Aggregate TDN	<0.01 (−0.01 to 0.01)	.76	<0.01 (−0.01 to 0.01)	.34	0.02 (0.01 to 0.04)	.01	<0.01 (−0.01 to 0.01)	.56
Aggregate TDO	−0.01 (−0.02 to 0.02)	.83	0.01 (−0.01 to 0.02)	.53	−0.01 (−0.05 to 0.03)	.65	<0.01 (−0.02 to 0.03)	.72

Abbreviations: BSMAS, Bergen Social Media Addiction Scale; GAD-7, Generalized Anxiety Disorder-7; ISI, Insomnia Severity Index; NA, not applicable; NSMCS, Negative Social Media Comparison Scale; PHQ-9, Patient Health Questionnaire-9; PSMU, problematic social media use; PUSNS, Problematic Use of Social Networks Scale; SMU, social media use; TDN, total daily notification; TDO, total days opened; TDP, total daily pickups; TDS, total daily screen time; UCLA-LS, University of California, Los Angeles Loneliness Scale.

^a^ *R*^2^ = 0.37 (95% CI, 0.29-0.44).

^b^ *R*^2^ = 0.30 (95% CI, 0.22-0.38).

^c^ *R*^2^ = 0.25 (95% CI, 0.18-0.33).

^d^ *R*^2^ = 0.39 (95% CI, 0.29-0.48).

^e^ Significance tested at α < .05 from 2-tailed tests.

### Detox Effects on Mental Health

Among the 295 participants who completed the 1-week detox intervention, significant reductions were observed for symptoms of depression (Cohen *d*, −0.37; 95% CI, −0.49 to −0.32; *P* < .001), anxiety (Cohen d, −0.44; 95% CI, −0.56 to −0.32; *P* < .001), and insomnia (Cohen *d*, −0.44; 95% CI, −0.56 to −0.32; *P* < .001), but not loneliness; see Table 3 for details. When stratified by depression clinical severity, the detox was more efficacious for participants with more severe depressive symptoms. This effect was most pronounced for participants with moderately severe symptoms with effect sizes of −0.97 (95% CI, −1.52 to −0.04) for depression, −0.84 (95% CI, −1.52 to −0.04) for anxiety, and −0.66 (95% CI,−1.15 to −0.16) for insomnia. Participants with no baseline depression showed smaller, but still significant, reductions in GAD-7 and ISI scores.

**Table 3.** Pre-Post Detox Changes in Mental Health Outcomes Stratified by Clinical Depression Severity

Variable	Mean (SD)			% Change, mean (SD)	*P* value^a^	Effect size (95% CI)
	Baseline	Postdetox	Change			
Complete detox intervention cohort						
CDC (n = 295)						
PHQ-9	5.95 (4.80)	3.90 (4.47)	−2.05 (4.63)	−24.81(78.59)	<.001	−0.44 (−0.56 to −0.32)
GAD-7	5.69 (4.72)	3.84 (4.51)	−1.89 (4.29)	−16.13 (84.74)	<.001	−0.44 (−0.56 to −0.32)
ISI	7.62 (5.32)	5.64 (5.59)	−1.97 (5.37)	−14.46 (96.54)	<.001	−0.37 (−0.49 to −0.25)
UCLA-LS	38.56 (11.56)	37.16 (12.16)	−0.40 (7.44)	0.34 (20.56)	.53	−0.05 (−0.17 to 0.06)
Stratified by depression clinical severity						
NOD (n = 153)						
PHQ-9	2.43 (1.50)	2.04 (2.21)	−0.39 (2.21)	−10.01 (96.73)	.08	−0.18 (−0.34 to −0.02)
GAD-7	2.97 (2.87)	2.33 (2.82)	−0.64 (2.63)	−3.54 (93.99)	.01	−0.24 (−0.40 to −0.08)
ISI	5.04 (3.93)	4.11 (4.28)	−0.94 (3.77)	−6.90 (111.37)	.01	−0.25 (−0.41 to −0.09)
UCLA-LS	33.62 (8.86)	33.25 (8.92)	−0.37 (7.00)	0.64 (21.32)	.57	−0.05 (−0.21 to 0.10)
MLD (n = 88)						
PHQ-9	7.18 (1.56)	4.82 (3.40)	−2.36 (3.45)	−31.48 (46.27)	<.001	−0.68 (−0.92 to −0.45)
GAD-7	7.27 (4.07)	4.70 (4.43)	−2.57 (4.60)	−22.21 (79.05)	<.001	−0.56 (−0.79 to −0.33)
ISI	8.99 (4.72)	6.87 (5.52)	−2.10 (5.61)	−14.37 (82.59)	<.001	−0.37 (−0.59 to −0.16)
UCLA-LS	40.72 (10.55)	40.10 (10.22)	−0.61 (7.80)	−0.38 (21.16)	.63	−0.08 (−0.29 to 0.13)
MRD (n = 32)						
PHQ-9	11.43 (1.44)	7.01 (6.30)	−4.42 (6.53)	−37.46 (55.37)	<.001	−0.68 (−1.05 to −0.30)
GAD-7	9.46 (3.59)	6.00 (5.59)	−3.46 (5.48)	−37.01 (58.01)	.001	−0.63 (−1.01 to −0.26)
ISI	11.22 (4.18)	7.34 (6.41)	−3.88 (6.81)	−30.87 (63.69)	.004	−0.57 (−1.36 to −0.31)
UCLA-LS	46.57 (11.12)	45.41 (13.05)	−1.17 (4.22)	−3.11 (9.67)	.14	0.09 (−0.03 to 0.21)
MSD (n = 18)						
PHQ-9	16.67 (2.61)	8.21 (7.87)	−7.98 (8.21)	−48.00 (60.61)	.001	−0.97 (−1.52 to −0.43)
GAD-7	12.12 (4.41)	6.49 (7.08)	−5.62 (6.72)	−48.40 (59.13)	.003	−0.84 (−1.36 to −0.31)
ISI	14.56 (5.84)	8.77 (8.33)	−5.79 (8.80)	−35.04 (73.70)	.02	−0.66 (−1.15 to −0.16)
UCLA-LS	51.48 (10.51)	51.83 (14.39)	0.35 (11.04)	1.73 (23.95)	.91	0.03 (−0.42 to 0.48)
SRD (n = 4)						
PHQ-9	21.88 (2.17)	9.38 (11.15)	−12.5 (10.11)	−58.92 (50.21)	.13	−1.24 (−2.54 to 0.07)
GAD-7	16.62 (6.55)	9.50 (11.09)	−7.12 (7.79)	−54.27 (53.26)	.21	−0.91 (−2.08 to 0.25)
ISI	17.25 (6.95)	9.50 (11.70)	−7.75 (9.64)	−54.31 (54.71)	.25	−0.80 (−1.93 to 0.32)
UCLA-LS	57.00 (13.20)	57.5 (14.46)	0.50 (5.40)	0.59 (9.67)	.87	−0.09 (−0.89 to 1.07)

Abbreviations: CDC, complete detox cohort; GAD-7, Generalized Anxiety Disorder-7; ISI, Insomnia Severity Index; MLD, mild depression; MRD, moderate depression; MSD, moderately severe depression; NOD, no depression; PHQ-9, Patient Health Questionnaire-9; SRD, severe depression; UCLA-LS, University of California, Los Angeles Loneliness Scale.

^a^ Significance tested at α < .05.

### Detox Effects on Behavior Change

To assess the outcomes of the 1-week detox intervention on behavior, we examined changes in digital phenotyping and EMA features (Table 4). Statistically significant changes were observed across screen use and mobility domains. Specifically, daily screen duration (social media and non-social media) increased by 4.5% (15.4; 95% CI, 4.86-25.90 seconds; *P* = .04), while home time increased by 6.3% (42.78; 95% CI, 24.31-61.23 minutes; *P* < .001) during detox compared with baseline. These outcomes, however, were modest relative to the large day-to-day variability observed, with the within-participant residual SD for home time being 347.6 minutes and 198.0 seconds for screen duration. In sensitivity analyses to account for potential spatial confounding, the detox effect estimates were essentially unchanged (eTable 4 in Supplement 1), with much of the within-participant variability unexplained by geographic clustering. No other changes were statistically significant across screen interaction, mobility entropy, communication, or EMA-based measures.

**Table 4.** Results of Random-Effects Model for Outcomes Before and After Social Media Detox Intervention

Outcome^a^	Social media detox intervention period, mean (SD)		β (95% CI)	Residual SD	Change, %	*P* value^b^
	Before	After				
Screen use						
Screen wakes	207.0 (137.4)	206 (134.8)	−0.40 (−6.74 to 5.95)	119.5	5.3	.92
Screen unlocks	103.7 (73.0)	106.0 (73.2)	2.32 (−0.91 to 5.55)	60.9	3.6	.36
Screen unlock duration, s	332.1 (204.6)	329.1 (212.2)	−2.85 (−12.47 to 6.77)	181.2	−0.1	.73
Screen duration, s	445.9 (233.2)	462.1 (238.0)	15.37 (4.86 to 25.90	198.0	4.5	.04
Mobility						
Home time, min	860.0 (374.5)	903.5 (354.4)	42.78 (24.31 to 61.23)	347.6	6.3	<.001
Entropy	0.5 (0.4)	0.5 (0.4)	−0.01 (−0.03 to 0.01)	0.37	1.7	.70
Step count	6484.0 (4751.9)	6590.8 (4925.9)	121.64 (−103.55 to 346.82)	4240.0	5.2	.49
Text						
Incoming texts, No.	107.9 (117.0)	112.3 (122.5)	4.66 (−0.88 to 10.21)	104.4	12.5	.34
Outgoing texts, No.	80.6 (97.7)	84.4 (103.3)	3.94 (−0.76 to 8.64)	88.4	18.2	.34
Text degree	46.4 (41.4)	47.6 (42.5)	1.35 (−0.56 to 3.27)	36.1	7.2	.36
Call						
Incoming calls, No.	2.8 (2.9)	2.9 (2.9)	0.08 (−0.06 to 0.23)	2.8	9.3	.49
Outgoing calls, No.	3.0 (3.7)	3.0 (3.9)	0.03 (−0.16 to 0.22)	3.6	10.5	.49
Call degree	3.0 (2.4)	3.0 (2.6)	0.01 (−0.12 to 0.13)	2.4	4.0	.92
Call duration, s	24.0 (56.2)	22.5 (37.9)	−0.90 (−3.47 to 1.67)	48.1	15.6	.70
EMAs						
Mood	3.0 (1.8)	2.9 (1.8)	−0.11 (−0.19 to −0.02)	1.6	−2.3	.06
Anxiety	2.7 (2.4)	2.8 (2.4)	0.07 (−0.03 to 0.18)	3.9	12.4	.36
Difficulty functioning	0.9 (0.9)	0.90 (0.90)	0.01 (−0.03 to 0.04)	0.7	2.5	.86

Abbreviation: EMA, ecological momentary assessment.

^a^ Outcomes are per day estimates.

^b^ Significance tested at α < .05 from 2-tailed tests.

## Discussion

By leveraging objective social media use data, our cohort study corroborates prior findings that metrics of social media use have small or no association with mental health outcomes in youth.^5,8^ Our findings also suggest that the problematic uses of social media, particularly those involving negative social comparison, are more consistently associated with greater symptom severity of depression, anxiety, and insomnia. In addition, our findings contribute to the growing body of evidence suggesting that brief digital detox interventions may offer meaningful mental health benefits,^38^ particularly among individuals with higher baseline symptom severity. By integrating smartphone-based digital phenotyping and EMAs, this study offers novel insights into potential behaviors associated with reduced social media use during detox, a dimension of use rarely captured in prior research.

Findings on the association between social media use and mental health outcomes have long been mixed.^1,2,3,4,5^ Our results align with the few studies that have used objective social media use data, showing little evidence for a robust association between overall use and mental health outcomes.^13^ In our sample, objective measures such as screen time, notifications, and pickups had small associations with symptoms of depression, anxiety, loneliness, and insomnia. In contrast, we observed positive associations between self-reported problematic or addictive social media use and mental health outcomes. These findings suggest that the impact of social media on the mental health of young adults may depend less on the quantity of use and more on the emotional and psychological state in which use occurs.

The 1-week social media detox intervention led to significant reductions in symptoms of depression, anxiety, and insomnia. Notably, these improvements were more pronounced among participants with higher baseline symptom severity. Participants with moderately severe depression experienced the greatest reductions across multiple symptom domains, suggesting that individuals with greater symptom burden may derive the most benefit from structured reductions in social media use.^39^ In contrast, symptoms of loneliness did not significantly improve, which may reflect the inherently social role of certain platforms, where reduced engagement could paradoxically diminish feelings of social connection and community. We speculate that the improvements observed during detox were associated more with a reduction in opportunities for problematic engagement, such as negative social comparison and addictive use,^40,41,42^ rather than by reductions in overall screen time, consistent with our findings showing lesser associations between objective screen time and mental health outcomes.

Although participants reduced their use of social media, they had, on average, longer screen durations and time spent at home during the detox period compared with the 2-week baseline. However, these findings occurred against a backdrop of substantial within-person variability in behavior, as indicated by the mixed-effects model residuals. To mitigate fluctuations in these passive feature estimates, future studies may benefit from including a trial or acclimation period before baseline to allow participants to adjust to study procedures.^43^ Furthermore, since our findings suggest that problematic social media use is more associated with adverse mental health outcomes than the quantity of use, interventions may be more effective if they target reducing problematic engagement rather than focusing exclusively on overall reduction in use.

### Strengths and Limitations

Our study has several notable strengths. First, the use of objective social media use measures provided a more accurate estimate of youth engagement and its association with problematic use and mental health outcomes. Second, we employed a multipronged approach that integrated digital phenotyping data, EMAs, and validated clinical measures to comprehensively capture changes in momentary states, behavior and mental health.

This study has several limitations. First, the sample was nonclinical and predominantly female, college-aged, iOS users, and highly educated. Second, our survey-based measures and EMAs relied on self-report and may be influenced by recall or social desirability bias. Although device-recorded app usage statistics cannot be altered by participants, they could be reset or circumvented by using alternate devices, and systematic differences between platforms (iOS vs Android) may introduce variability in screen time estimates. Third, participant behavior may have been influenced by reactivity to being monitored, and self-selection into the detox group may reflect underlying differences in motivation or symptom severity that limit causal inference.

Fourth, although we addressed missing data using MICE after evaluating the plausibility of the missing at random assumption with the Hawkins test, this assumption may not fully hold in practice, as sensor data can be missing due to behaviors linked to unobserved states (eg, disengaging from device use during mood changes) as well as random factors such as hardware malfunction, battery depletion, or connectivity issues. Fifth, the study had no follow-up period, precluding conclusions about the long-term durability of effects, and the absence of a randomized control group means improvements in mental health outcomes cannot be definitively attributed to the intervention. In addition, the detox may be susceptible to demand effects, as participants were compensated and may have been aware of the expected outcome.

## Conclusions

In this cohort study, a 1-week social media detox led to significant reductions in symptoms of depression, anxiety, and insomnia, particularly among individuals with greater baseline symptom severity. These findings suggest that digital behavior change interventions may improve mental health; however, the durability of these outcomes and their impact on behavior warrant further study, particularly in a more diverse population.

## Notes

Supplement 1. eMethods. eFigure 1. Social Media Study Diagram eFigure 2. Screenshots From the mindLAMP Application eFigure 3. Example Digital Data Summary Report eFigure 4. Example of iOS App Usage Phone Settings Interface eFigure 5. Example of Android App Usage Phone Settings Interface eFigure 6. Study Flow Chart eFigure 7. Social Media Usage During the Baseline Period eFigure 8. Mean Daily Social Media Screen Time by Baseline Characteristics eFigure 9. Adherence to Social Media Detox by Platform eTable 1. Baseline Clinical Characteristics eTable 2. Baseline Correlations Among PSMU and SMU Features eTable 3. Associations Among Self-Reported PSMU, Objective SMU and Mental Health Outcomes eTable 4. Gaussian Process Regression Model Estimates of Spatial Variation Across Outcomes

Supplement 2. Data Sharing Statement
