# Supplementary material for: Social Media Detox and Youth Mental Health
Source: JAMA Netw Open. 2025 Nov 24;8(11):e2545245. doi: 10.1001/jamanetworkopen.2025.45245 (PMC12645342; doi:10.1001/jamanetworkopen.2025.45245)
Supplement: Supplement 1. — eMethods. eFigure 1. Social Media Study Diagram eFigure 2. Screenshots From the mindLAMP Application eFigure 3. Example Digital Data Summary Report eFigure 4. Example of iOS App Usage Phone Settings Interface eFigure 5. Example of Android App Usage Phone Settings Interface eFigure 6. Study Flow Chart eFigure 7. Social Media Usage During the Baseline Period eFigure 8. Mean Daily Social Media Screen Time by Baseline Characteristics eFigure 9. Adherence to Social Media Detox by Platform eTable 1. Baseline Clinical Characteristics eTable 2. Baseline Correlations Among PSMU and SMU Features eTable 3. Associations Among Self-Reported PSMU, Objective SMU and Mental Health Outcomes eTable 4. Gaussian Process Regression Model Estimates of Spatial Variation Across Outcomes [file jamanetwopen-e2545245-s001.pdf]

Supplemental Online Content

Calvert E, Cipriani M, Dwyer B, et al. Social media detox and youth mental health. *JAMA Netw Open*. 2025;8(11):e2545245. doi:10.1001/jamanetworkopen.2025.45245

eMethods.

eFigure 1. Social Media Study Diagram

eFigure 2. Screenshots From the mindLAMP Application

eFigure 3. Example Digital Data Summary Report

eFigure 4. Example of iOS App Usage Phone Settings Interface

eFigure 5. Example of Android App Usage Phone Settings Interface

eFigure 6. Study Flow Chart

eFigure 7. Social Media Usage During the Baseline Period

eFigure 8. Average Daily Social Media Screentime by Baseline Characteristics

eFigure 9. Adherence to Social Media Detox by Platform

eTable 1. Baseline Clinical Characteristics

eTable 2. Baseline Correlations Among PSMU and SMU Features

eTable 3. Relationships Among Self-Reported PSMU, Objective SMU and Mental Health Outcomes

eTable 4. Gaussian Process Regression Model Estimates of Spatial Variation Across Outcomes

This supplemental material has been provided by the authors to give readers additional information about their work.

© 2025 Calvert E et al. *JAMA Network Open*.

© 2025 Calvert E et al. *JAMA Network Open*.

eMethods

Participant Recruitment

Posts on ResearchMatch included a link to schedule a virtual visit via Zoom with a research assistant. Contact information for the study coordinators was also provided in case prospective participants had any questions. If a participant called the contact number, a brief screening was conducted to confirm interest in the study. ResearchMatch.org is a national, electronic, web-based recruitment tool created through the Clinical & Translational Science Awards Consortium in 2009 and is maintained at Vanderbilt University. There is no cost for researchers at participating institutions in the ResearchMatch Network to use ResearchMatch for feasibility analysis or recruitment. Additionally, the team's Instagram page was used for recruitment, posting an advertisement with an IRB-approved message that included information on study procedures, remuneration, a link to schedule the first appointment, and contact information.

Detailed Study Procedure

Study Visit 1: This visit was scheduled to last one hour. Participants were asked to confirm their name, age, phone number, and current location for safety reasons. They were then informed that they could earn up to $150, which was dependent on the number of surveys completed throughout the study (US $7 per daily EMA survey). The Research Assistant reviewed the written informed consent form with the participant, assessing their understanding through open-ended questions. Informed written consent was obtained through the Research Electronic Data Capture (REDCap) e-signature process. Participants then completed the first battery of standard psychological questionnaires using a secure REDCap account. These included the Patient Health Questionnaire-9 (PHQ-9), Generalized Anxiety Disorder-7 (GAD-7), UCLA Loneliness Scale, Bergen Social Media Addiction Scale (BSMAS), Problematic Use of Social Networks Scale (PUSNS), Negative Social Media Comparison Scale (NSMCS), Rosenberg Self-Esteem Scale (RSES), Insomnia Severity Index (ISI), and demographic questions (further details on these scales are provided in the 'Measures' section below). If any of the scales indicated clinical risk that required immediate attention, the Research Assistant notified the Principal Investigator (PI), who conducted a safety assessment. If risk was detected, the PI alerted local authorities, including for scores ≥2 on question 9 of the PHQ-9. Participants were then guided through downloading and logging into the mindLAMP app, as well as how to access and complete the necessary surveys. They were instructed to complete daily and weekly surveys, with details provided in the Measures section below. Safety plans were established in case a participant scored higher than 2 on question 9 of the PHQ-9.

Between Visit 1 and Visit 2: The app will remind participants to complete their daily surveys over the course of the 2-week period. Passive data, including GPS, accelerometer, phone use data, and SensorKit data, will be continuously collected. Participants will also receive the same battery of assessments to complete, excluding the demographic questions via REDCap.

Study Visit 2: Two weeks after their intake visit, participants attended another virtual meeting with a Research Assistant. They completed the same battery of assessments as during the first visit, excluding the demographic questions. The Research Assistant shared the participant's data report and assisted in summarizing and interpreting the data. Participants were then asked to report their

time spent on each social media app over the past two weeks, based on the information from their Settings app. This was recorded in REDCap. If a participant decided to end the study, they were instructed to delete the mindLAMP app to deactivate the data stream coming from their phone, and their financial compensation would be sent via ClinCard. If the participant chose to continue with the study, they were asked to refrain from social media use on all across Facebook, Instagram, Snapchat, TikTok, and Twitter/X for the next 7 days and continue completing daily and weekly surveys as previously instructed.

Study Visit 3: Participants who opted into the detox attended their final virtual meeting one week after their second visit (Visit 2). They completed the same battery of assessments as during the second visit. The Research Assistant shared the final data report with the participant, assisting in summarizing and interpreting the data (figure S1). Participants were then asked to report their app-level usage data for each social media app over the detox week, based on information from their Settings app (figure S2) . This was recorded in REDCap. Participants were instructed to delete the mindLAMP app to deactivate the data stream from their phone, and we also deactivated their account to ensure no further data collection.

Data Privacy: None of the data collected from call and text logs or social media usage included phone numbers, nor did it record or contain any content from phone calls or text messages. Only the time and duration of calls, texts, and social media use were recorded and used in data analysis. For all GPS data collected, the analysis focused on detecting changes from each participant's personal baseline of mobility, and no exact location data was used or reported. For example, we analyzed whether a participant was more active or traveled further than usual on certain days, but we did not track or report their specific locations.

Passive Data Derivation

Passive Smartphone Sensors
By utilizing the mindLAMP application developed at the Beth Israel Deaconess Medical Center (see details on pp 8), data from the following smartphone sensors were collected for each patient in the study.

Accelerometer: The mindLAMP app recorded the phone's accelerometer data, which tracked the movement of the phone. This data can be used to estimate how long a participant remained still, when they were walking, and how many steps they took while walking. It is important to note that all smartphones now automatically collect this data as part of their built-in step tracking features.

GPS: Several times an hour, the mindLAMP app recorded the phone's GPS location, capturing latitude and longitude, along with the precision of that measurement. The GPS is typically accurate within about 10-20 meters. It can be used to construct a map of where a participant traveled and when they were at different locations, though it cannot identify the mode of travel. It is important to note that all smartphones automatically collect this data unless turned off by the user. Since our analysis focused only on changes in mobility, as indicated by GPS, we never reported the specific locations or places where participants went or spent time. Instead, we focused on metrics such as the number of locations visited per day and the amount of distance traveled per day.

Phone/Screen Usage: The mindLAMP app recorded when the participant turned the phone's screen on or off, when the phone was rebooted, and when it was plugged in or unplugged. Tracking screen activity serves as a proxy for phone usage. For example, if a participant woke up at 3:44am,

© 2025 Calvert E et al. *JAMA Network Open.*

checked their phone for 10 seconds, and then went back to sleep, the app would record that the phone screen was on for 10 seconds at 3:44am.

Call/Text Logs: Call and text log data indicate when a phone call was made or a text message was sent, along with the duration of each interaction. The app does not collect any content from the call or text message, so no information was recorded regarding what was said or typed. The app does record the phone number dialed but encrypts it to ensure that the actual phone number remains confidential.

Derived Passive Features
Passively sensed smartphone data were collected daily and transformed into interpretable secondary features using the open-source Python library Cortex, developed by the Division of Digital Psychiatry at Beth Israel Deaconess Medical Center. Mobility features included Hometime (hours spent at home per day), Entropy (a 0–1 index capturing the diversity of locations visited), and Step Count (total steps per day). Screen interaction features included Screen Duration (total seconds the screen was active), Screen Wakes (number of times the screen was activated), Screen Unlocks (number of times the phone was unlocked), and Screen Unlock Duration (total seconds the screen remained unlocked). Communication features included Text Degree (number of unique contacts texted per day), Outgoing Text Number (texts sent per day), Incoming Text Number (texts received per day), Call Degree (unique contacts called per day), Outgoing Call Number (calls made per day), and Incoming Call Number (calls received per day).

Survey-based Measures

The 9-item Patient Health Questionnaire (PHQ-9) was used to assess the presence of depressive symptoms in participants over the past two weeks, both at baseline and after the detox intervention. Responses ranged from 0 (not at all) to 3 (nearly every day), with a total score range of 0-27. Clinical improvement was defined as a reduction of 4 or more points in a participant's score.

The 7-item Generalized Anxiety Disorder (GAD-7) scale was used to assess the presence of anxiety symptoms in participants over the past two weeks, at both baseline and after the detox intervention. Responses ranged from 0 (not at all) to 3 (nearly every day), with a score range of 0-21. Clinical improvement was indicated by a reduction of 4 or more points in a participant's score.

The 20-item University of California, Los Angeles Loneliness Scale (UCLA-LS) was used to evaluate feelings of loneliness and social isolation at baseline and after the detox intervention. Responses ranged from 1 (never) to 4 (often), with a total score range of 20-80. Higher scores indicated greater feelings of loneliness.

The 7-item Insomnia Severity Index (ISI) was used to assess the nature and severity of participants' sleep problems. Each item is rated on a scale from 0 to 4, with varying descriptions for each. For item 1, the subitems are rated from 0 (none) to 4 (very), while item 2 ranges from 0 (very satisfied) to 4 (very dissatisfied). Item 3 ranges from 0 (not at all interfering) to 4 (very much interfering), item 4 from 0 (not at all noticeable) to 4 (very much noticeable), and item 5 from 0 (not at all) to 4 (very much). Scores across all items are summed, with the total score ranging from 0 to 28 , where higher scores indicate greater insomnia severity.

The 10-item Rosenberg Self-Esteem Scale (RSES) was used to assess global self-worth by measuring both positive and negative feelings about oneself. For items 3, 5, 8, 9, and 10, responses

ranged from 0 (strongly agree) to 3 (strongly disagree), while for items 1, 2, 4, 6, and 7, responses ranged from 0 (strongly disagree) to 3 (strongly agree). The possible score range was 0-30, with higher scores indicating greater self-esteem.

The 18-item Problematic Use of Social Networking Scale (PUSNS) was used to evaluate participants' comparative use of social media and related addictive tendencies. Responses ranged from 1 (completely disagree) to 5 (completely agree), with a total score range of 18-90. Higher scores indicated more problematic social media use.

The 6-item Bergen Social Media Addiction Scale (BSMAS) was used to assess social media addiction among participants. Responses ranged from 1 (very rarely) to 5 (very often) to questions such as "Do you feel an urge to use social media more and more?" and "Do you use social media to forget about personal problems?". The total score range was 6-30, with higher scores reflecting greater social media addiction.

The 8-item Negative Social Media Comparison Scale (NSMCS) was used to assess how negatively participants compared themselves to others on social media. Responses ranged from 1 (strongly disagree) to 6 (strongly agree) to statements such as "I feel like other people's lives are better than mine" and "I feel like other people are more attractive than I am." The possible score range was 8-48, with higher scores indicating greater negative social media comparison.

EMA-based Measures

Anxiety: Consisted of a single question sent daily to participants in the study, "Overall, how would I rate my anxiety today?", rated from 0 (no anxiety) to 10 (worst it has ever been), with higher scores indicating greater feelings of anxiety.

Mood: Consisted of a single question sent daily to participants in the study, "Overall, how would you rate your mood today?", rated from 1 (worst) to 10 (best), The scale was inversed scored, with higher scores indicated worse mood.

Difficulty Functioning: Consisted of a single statement sent daily to participants in the study, "Please rate how strongly you agree or disagree with this statement: I am able to manage my day-to-day life", rated from 0 (strongly disagree) to 4 (strongly agree). The scale was inversed scored, so higher scores indicated higher difficulty functioning.

Statistical Analysis

Accounting for spatial confounding involved using a Bayesian Gaussian process regression with a Matérn kernel, where participants' home GPS coordinates (latitude and longitude) were included as inputs to the Gaussian process to model the latent spatial variation in our behavioral features. For each outcome feature Y_{it} measured for participant *i* on day *t:*

$$Y_{it} = \beta_0 + \beta_1 \,\mathbf{Period}_{it} + \beta_2 \,\mathbf{Age}_i + \beta_3 \,\mathbf{Gender}_i + \beta_4 \,\mathbf{Education}_i + \beta_5 \,\mathbf{Race}_i + \beta_6 \,\mathbf{PhoneType}_i + u_{0i} + f(longitude_i, latitude_i) + \epsilon_{it}$$

where:

- $f(longitude_i, latitude_i)$ is the spatial Gaussian process term with Matérn kernel
- β_0 is the fixed intercept
- u_{0i} is a participant-specific random intercept
- ϵ_{it} is the residual error

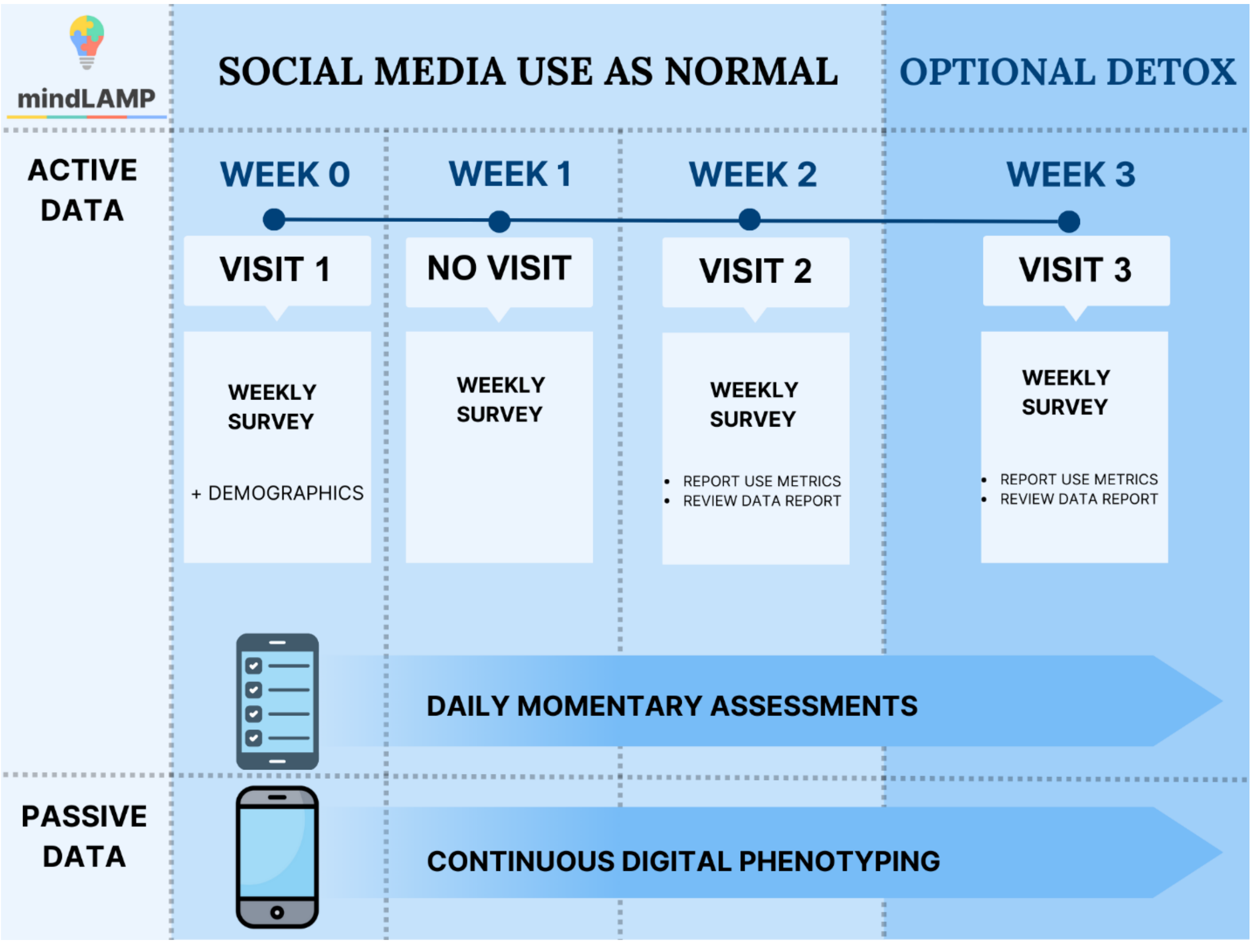

eFigure 1. Social Media Study Diagram

Overview of study protocol illustrating data collection across three visits using the mindLAMP mobile application. Participants completed active (daily EMAs and weekly surveys) and passive (digital phenotyping) data collection while maintaining normal social media use (Instagram, TikTok, Snapchat, Facebook, Twitter/X). An optional third visit involved a digital detox period, during which participants refrained from social media use.

© 2025 Calvert E et al. *JAMA Network Open*.

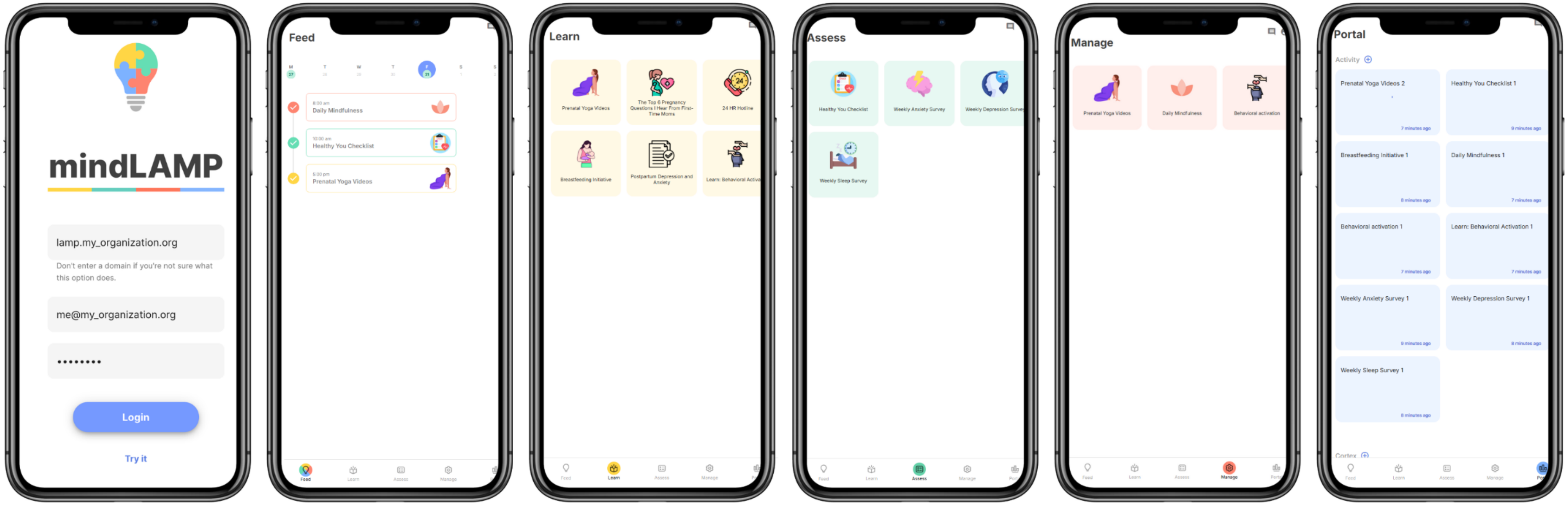

eFigure 2. Screenshots From the mindLAMP Application
mindLAMP is an open-source smartphone application developed by the Division of Digital Psychiatry at Beth Israel Deaconess Medical Center. The login page of mindLAMP that requires a user email and password to gain access to the app. The feed page that gives patients updates on surveys and activities they need to complete. The **L**earn tab contains psychoeducation modules. The **A**ssess tab contains questionnaires, surveys, and cognitive games to be completed by patients. The **M**anage tab contains CBT modules assigned to patients. The **P**ortal tab displays the collected patient data.

© 2025 Calvert E et al. *JAMA Network Open*.

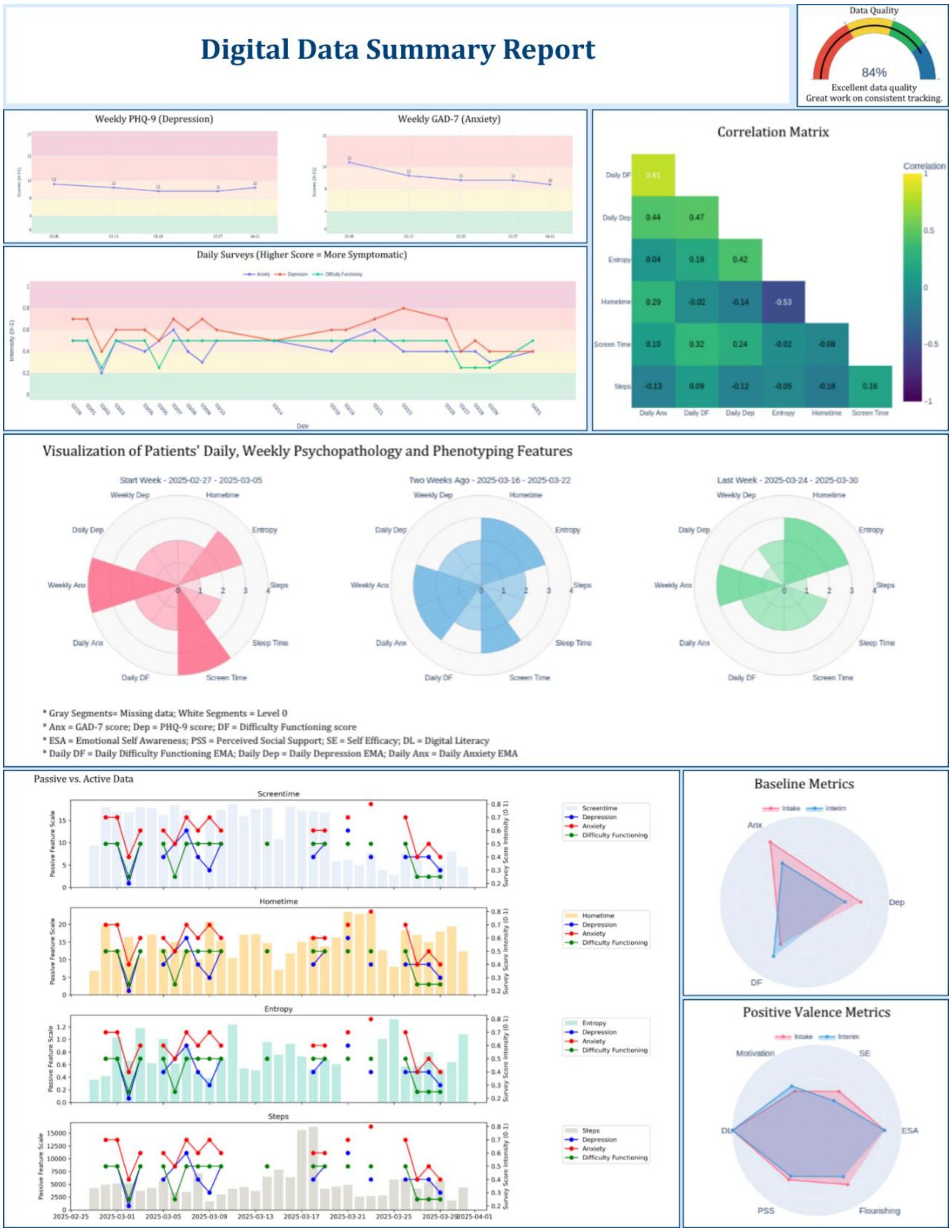

eFigure 3. Example Digital Data Summary Report: A comprehensive overview of weekly psychological measures (PHQ-9 for depression and GAD-7 for anxiety), daily EMA survey responses, and passive sensing data, including Screen Time, Hometime, Entropy, and Step Count. This data summary report is reviewed with patients at Visit 2 and Visit 3 if they opted into the detox intervention. The report includes visualizations of participants' daily and weekly psychopathology and phenotyping features, a correlation matrix, and comparisons of passive versus active data. Baseline and positive valence metrics are also presented, illustrating the relationship between self-reported and sensor-derived data.

© 2025 Calvert E et al. *JAMA Network Open*.

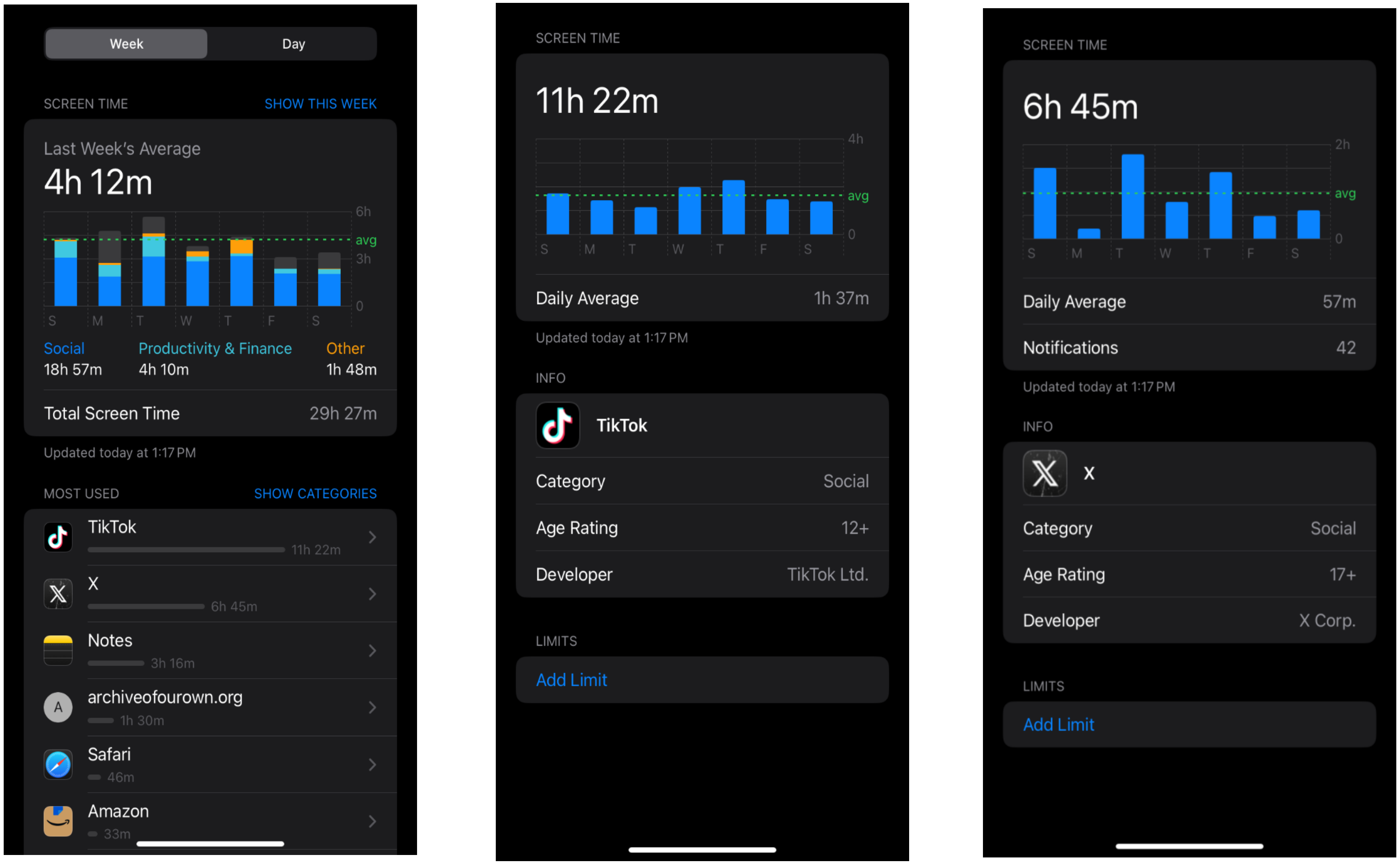

eFigure 4. Example of iOS App Usage Phone Settings Interface

At Visits 2 and 3, participants navigated to their device settings to access app usage data. Metrics recorded included overall screen time, as well as app-level screentime, notifications, pickups, and number of days opened for the five targeted social media platforms (Facebook, Instagram, Snapchat, TikTok, and Twitter/X). For each metric, both the mean daily estimate and cumulative totals over the two-week baseline period were documented.

© 2025 Calvert E et al. *JAMA Network Open*.

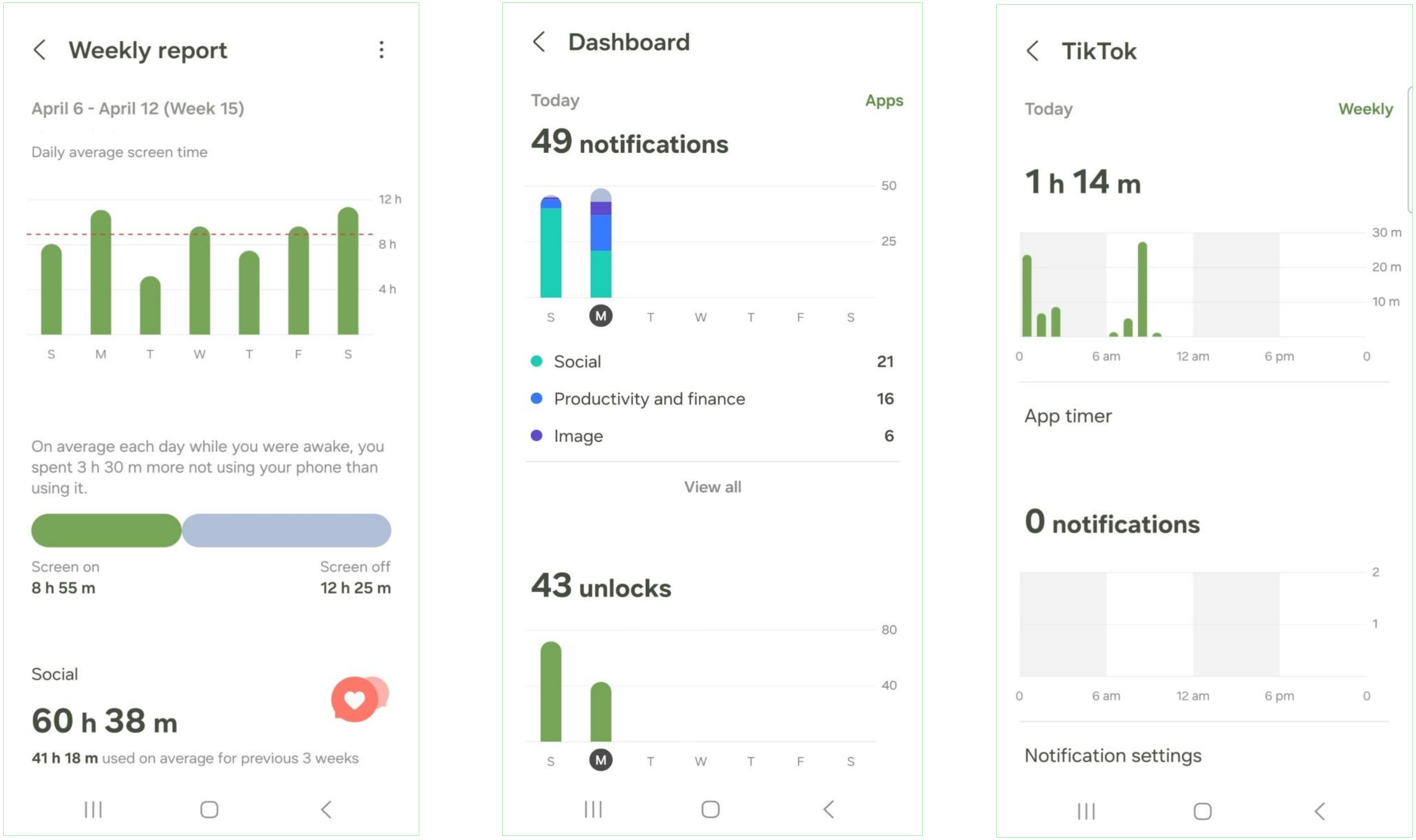

eFigure 5. Example of Android App Usage Phone Settings Interface
At Visits 2 and 3, participants navigated to their device settings to access app usage data. Metrics recorded included overall screen time, as well as app-level screentime, notifications, pickups, and number of days opened for the five targeted social media platforms (Facebook, Instagram, Snapchat, TikTok, and Twitter/X). For each metric, both the mean daily estimate and cumulative totals over the two-week baseline period were documented.

© 2025 Calvert E et al. *JAMA Network Open*.

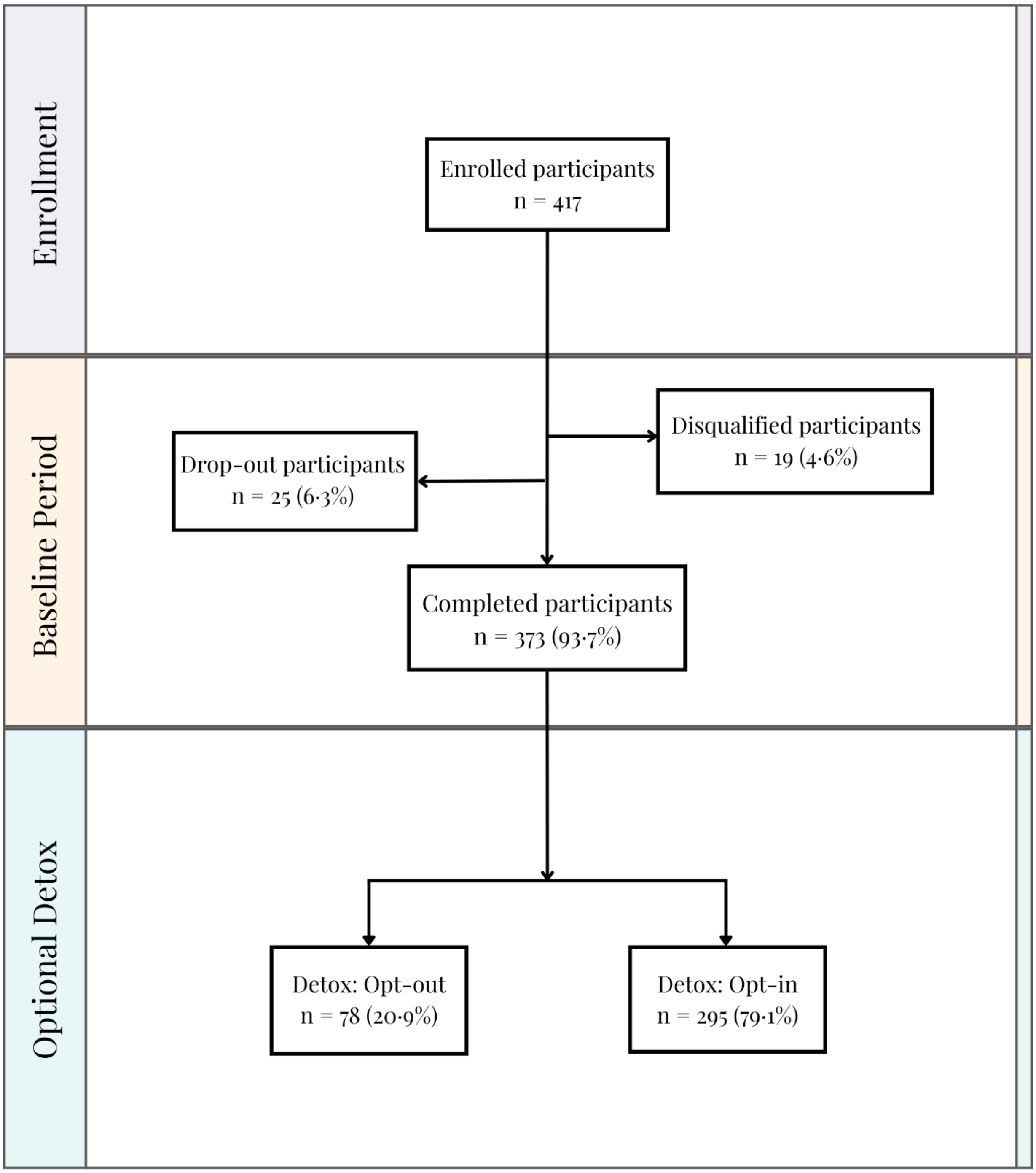

eFigure 6. Study Flow Chart

Flowchart depicting participant enrollment, attrition during the study period, and subsequent selection into the optional digital one-week detox phase of the study. Percentages indicate the proportion relative to the preceding participant group.

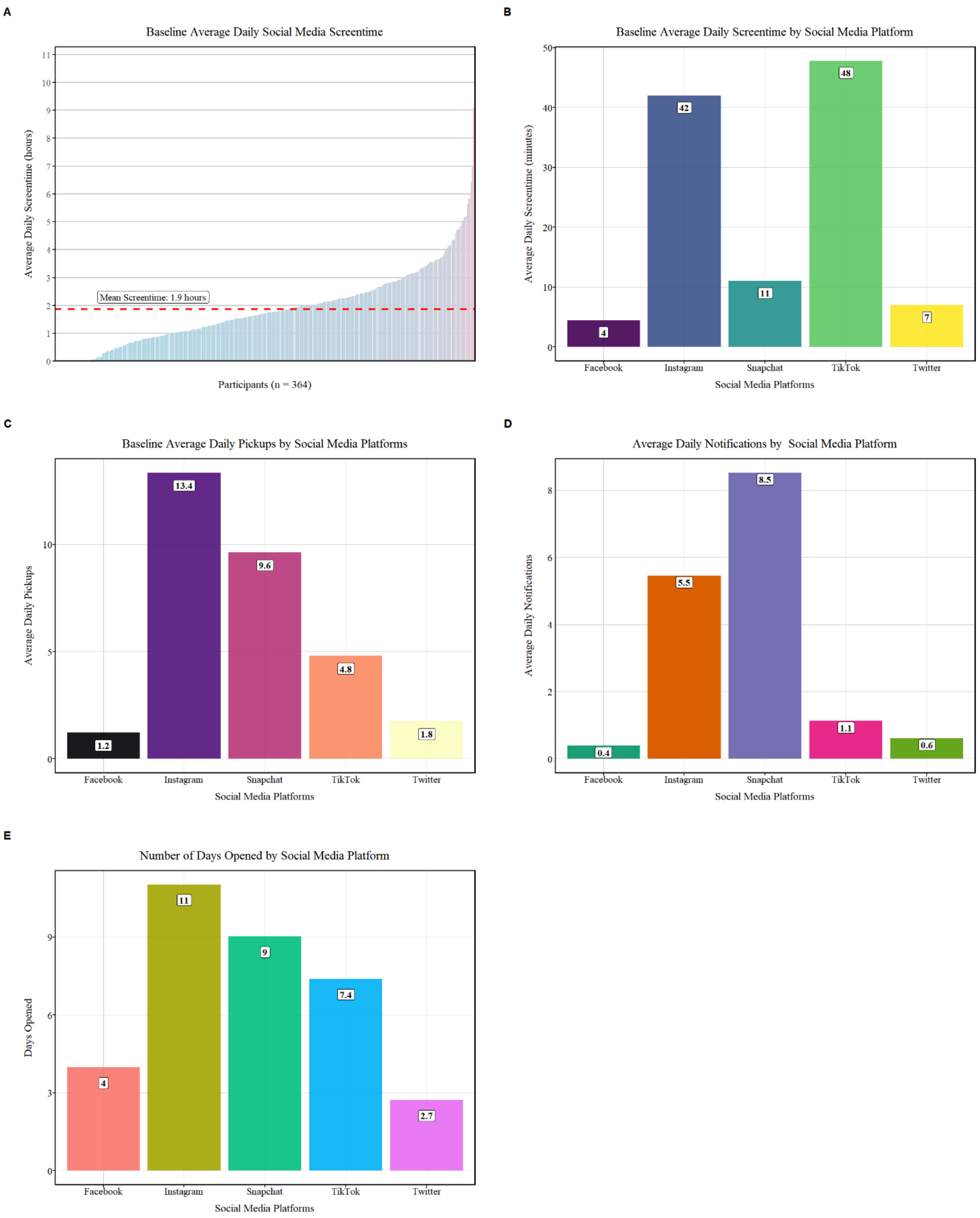

eFigure 7. Social Media Usage During the Baseline Period
(**A**) Distribution of participants' average daily social media screentime, highlighting mean usage; (**B**) Average daily screentime (in minutes) across individual social media platforms, with TikTok and Instagram being most used; (**C**) Average daily number of pickups per social media platform; (**D**) Average daily social media notifications received per platform; (**E**) Average number of days participants opened each social media platform during the two-week baseline.

© 2025 Calvert E et al. *JAMA Network Open.*

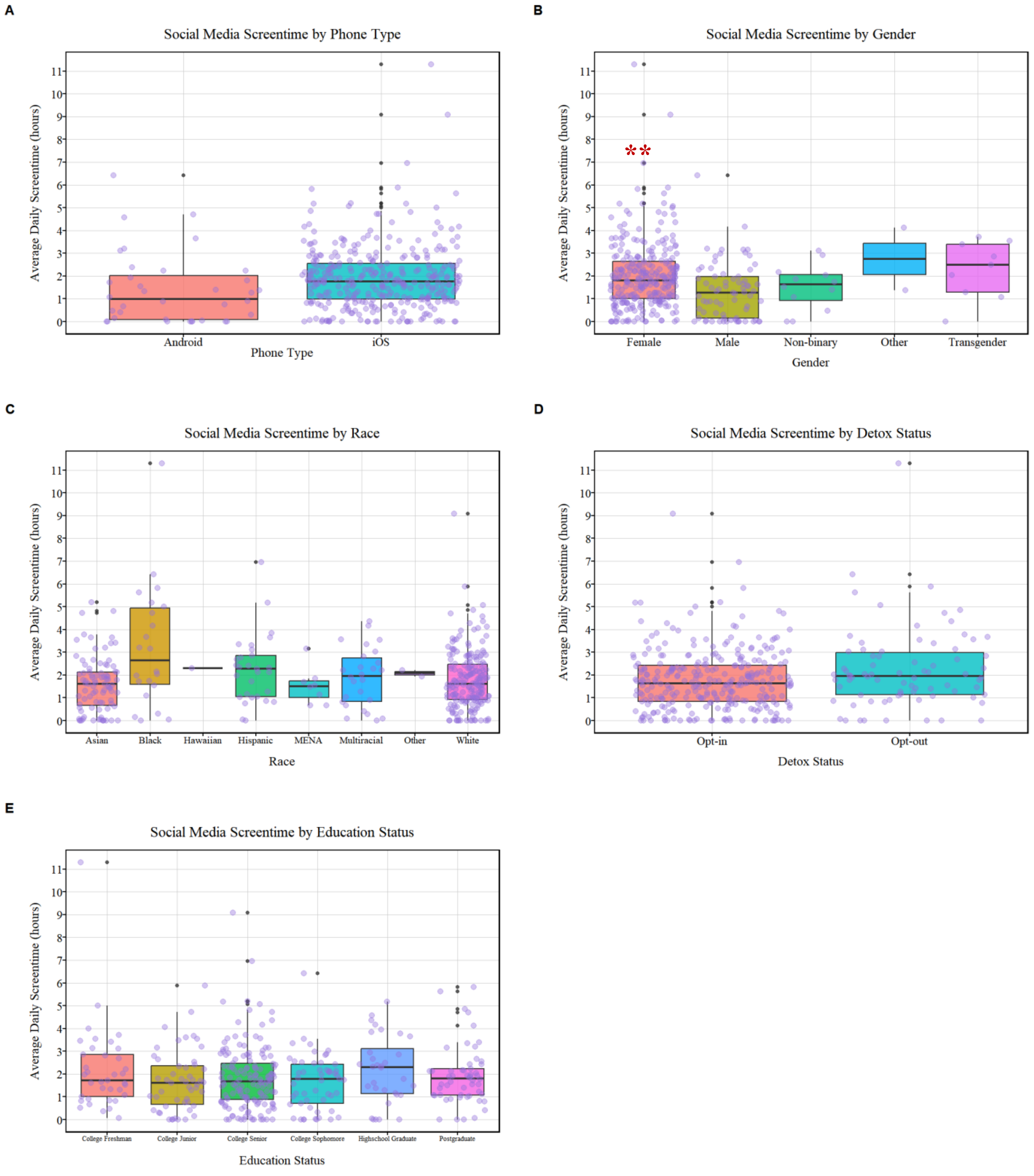

eFigure 8. Average Daily Social Media Screentime by Baseline Characteristics
Boxplots illustrating average daily social media screentime stratified by (**A**) Phone Type, (**B**) Gender, (**C**) Race, (**D**) Detox participation status (Opt-in vs. Opt-out), and (**E**) Education level. Individual data points are shown as dots. There were no statistically significant differences observed between groups across these categories.

Mean daily screentime was higher in female participants (2.0 hours [SD 1.5]) than in male participants (1.3 hours [SD 1.2]; $p<0.001$), with no other significant differences observed by race, educational status, mobile operating system, or detox opt-in status.

© 2025 Calvert E et al. *JAMA Network Open*.

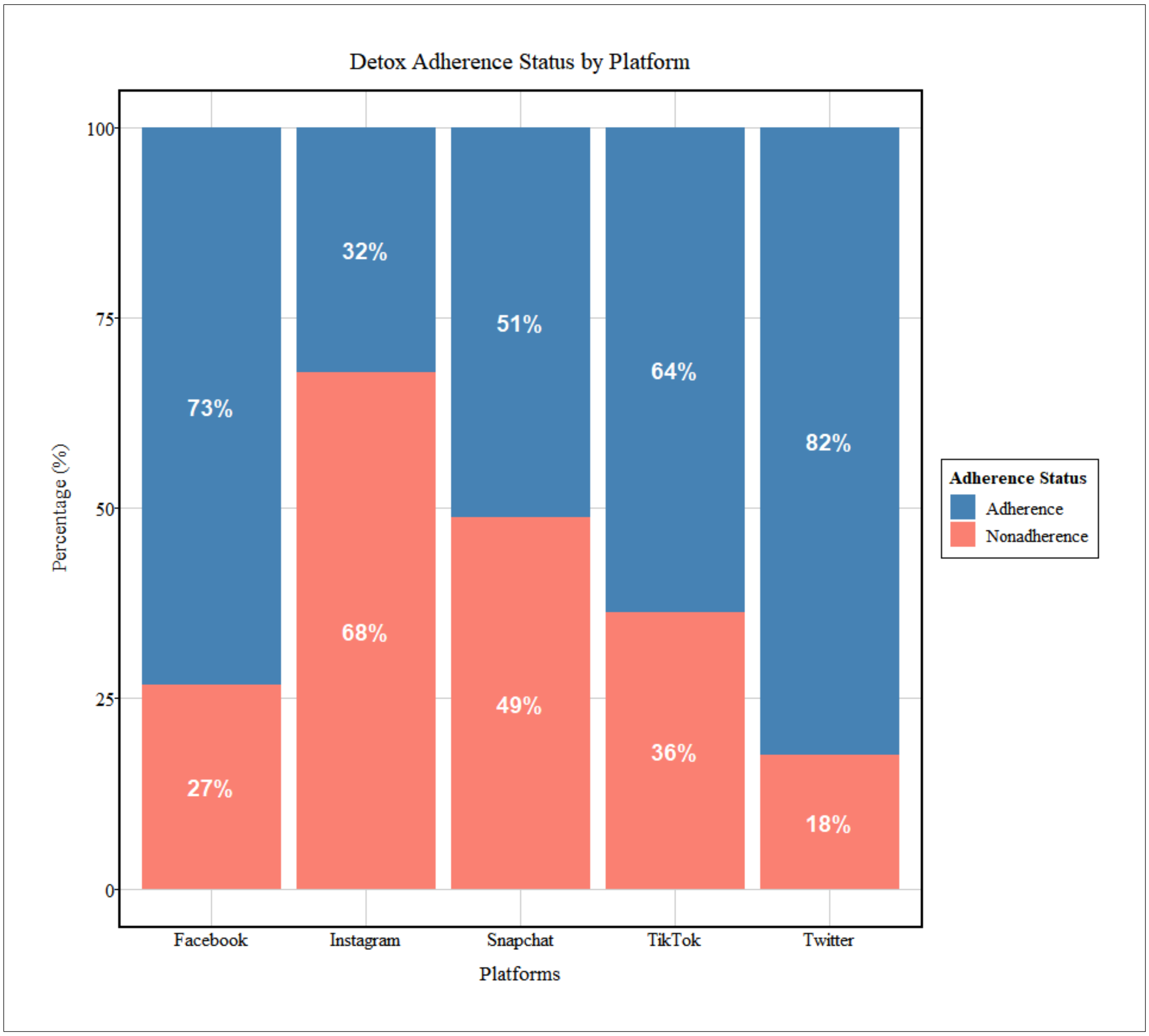

eFigure 9. Adherence to Social Media Detox by Platform
Percentage of participants who adhered to (blue) versus did not adhere to (red) detox guidelines, presented separately for each social media platform. Adherence rates were highest for Twitter (82.4%) and lowest for Instagram (32.2%).

© 2025 Calvert E et al. *JAMA Network Open*.

eTable 1. Baseline Clinical Characteristics (Median, IQR, Minimum, Maximum)

	PHQ-9	GAD-7	ISI	UCLA-LS	RSES	NSMCS	PUSNS	BSMAS
median	4	4	6	50	23	27	48	13
IQR	6	6	7	9	2	10	18	7
min	0	0	0	0	10	6	18	6
max	25	21	26	80	29	42	85	28

Abbreviations: Depression as assessed by the Patient Health Questionnaire-9 (PHQ-9); Anxiety as assessed by the General Anxiety Disorder-7 (GAD-7); Insomnia as assessed by the Insomnia Severity Index (ISI); Loneliness as assessed by the UCLA Loneliness Scale (UCLA-LS); Self-esteem as assessed by the Rosenberg Self-Esteem Scale (RSES); Social comparison as assessed by the Negative Social Media Comparison Scale (NSMCS); Problematic use as assessed by the Problematic Use of Social Networks Scale (PUSNS); Social media dependence as assessed by the Bergen Social Media Addiction Scale (BSMAS).

eTable 2. Baseline Correlations Among PSMU and SMU Features

			95% Confidence Interval		
**Variable 1**	**Variable 2**	**r**	**Lower**	**Upper**	***P* value[a]**
BSMAS	ADS Instagram	0.25	0.15	0.34	<0.01
BSMAS	ADS TikTok	0.16	0.06	0.26	0.02
PUSNS	ADS TikTok	0.14	0.04	0.24	0.04
BSMAS	TDS	0.32	0.23	0.41	<0.01
PUSNS	TDS	0.24	0.14	0.33	<0.01
BSMAS	ADN Instagram	0.20	0.10	0.30	<0.01
BSMAS	TDN	0.17	0.07	0.27	0.01
PUSNS	TDN	0.16	0.06	0.26	0.01

Abbreviations: PSMU, Problematic social media use; SMU, Social media use; BSMAS, Bergen Social Media Addiction Scale; PUSNS, Problematic Use of Social Networks Scale; NSMCS, Negative Social Media Comparison Scale; ADS, Average daily screentime; TDS, Total daily screentime; ADP, Average daily pickups; TDP, Total daily pickups; ADN, Average daily notifications; TDN, Total daily notification.

[a] Correlation coefficients that are significant after Benjamini-Hochberg correction.

© 2025 Calvert E et al. *JAMA Network Open.*

eTable 3. Relationships Among Self-Reported PSMU, Objective SMU and Mental Health Outcomes

	PHQ-9	GAD-7	UCLA-LS	ISI
**Self-reported PSMU**				
BSMAS	**0.47**[b]	**0.35**[b]	**0.27**[b]	**0.35**[b]
PUSNS	**0.48**[b]	**0.41**[b]	**0.43**[b]	**0.33**[b]
NSMCS	**0.45**[b]	**0.44**[b]	**0.49**[b]	**0.28**[b]
**Objective SMU**				
Screentime				
**TDS**	0.10	<-0.01	-0.01	**0.15**[b]
ADS Facebook	0.07	0.06	0.06	0.12[a]
ADS Instagram	0.10	0.01	-0.01	0.13[b]
ADS Snapchat	0.02	0.02	-0.05	0.05
ADS TikTok	0.01	-0.04	-0.02	0.05
ADS Twitter/X	0.09	0.04	0.05	0.07
Pickups				
**TDP**	0.03	<-0.01	-0.03	0.05
ADP Facebook	0.07	0.09	0.13[b]	0.10
ADP Instagram	<0.01	-0.03	-0.06	0.03
ADP Snapchat	<0.01	-0.03	-0.06	0.03
ADP TikTok	0.06	0.02	<0.01	0.04
ADP Twitter/X	0.05	0.04	0.07	0.04
Notifications				
**TDN**	0.07	0.06	0.11[a]	0.10
ADN Facebook	0.06	0.06	-0.01	0.04
ADN Instagram	**0.15**[b]	0.11[a]	0.08	**0.15**[b]
ADN Snapchat	-0.05	-0.02	0.02	<-0.01
ADN TikTok	0.03	0.01	-0.04	0.05
ADN Twitter/X	0.09	0.07	0.14[b]	0.10
Days Opened				
**TDO**	<0.01	0.03	-0.04	0.05
DO Facebook	0.12[a]	0.09	**0.15**[b]	0.13[a]
DO Instagram	0.01	0.01	-0.07	0.07
DO Snapchat	-0.05	0.03	-0.04	<-0.01
DO TikTok	-0.03	-0.05	-0.11[a]	0.03
DO Twitter/X	0.09	0.04	0.03	0.02

Abbreviations: PSMU, Problematic social media use; SMU, Social media use; BSMAS, Bergen Social Media Addiction Scale; PUSNS, Problematic Use of Social Networks Scale; NSMCS, Negative Social Media Comparison Scale; ADS, Average daily screentime; TDS, Total daily screentime; ADP, Average daily pickups; TDP, Total daily pickups; ADN, Average daily notifications; TDN, Total daily notification; DO, Days opened; TDO, Total days opened.
[a]*P* <0.05 (2-tailed)
[b]*P* <0.01 (2-tailed)
Boldface depicts correlation coefficients that are significant after Benjamini-Hochberg correction.

© 2025 Calvert E et al. *JAMA Network Open.*

eTable 4. Gaussian Process Regression Model Estimates of Spatial Variation Across Outcomes

		Spatial Gaussian Model Process Terms		
**Outcome**	**B (95% CI)**	**sdgp[a] (95% CI)**	**lscale[b] (95% CI)**	**sigma[c] (95% CI)**
Hometime	41.7 (23.1, 60.1)	237.6 (7.5, 854.7)	0.1 (0.01, 0.36)	347.7 (341.5, 354.1)
Entropy	-0.01 (-0.03, 0.01)	0.62 (0.01, 2.56)	0.11 (0.01, 0.64)	0.37 (0.36, 0.38)
Step count	125.1 (-104.6, 346.4)	3706.0 (128.7, 12684.6)	0.1 (0.01, 0.46)	4240.0 (4162.9, 4328.3)
Screen duration	14.9 (4.4, 25.4)	221.4 (7.4, 751.5)	0.01 (0.01, 0.38)	197.8 (194.3, 201.4)

B refers to the unstandardized coefficient (point estimate)
[a] Spatial variation standard deviation
[b] Length scale of the Gaussian process
[c] Residual variance standard deviation

© 2025 Calvert E et al. *JAMA Network Open.*
