# Supplementary material for: Social Media Detox and Youth Mental Health
Source: JAMA Netw Open. 2025 Nov 24;8(11):e2545245. doi: 10.1001/jamanetworkopen.2025.45245 (PMC12645342; doi:10.1001/jamanetworkopen.2025.45245)
Supplement: Supplement 2. — Data Sharing Statement [file jamanetwopen-e2545245-s002.pdf]

Data Sharing Statement

Calvert. Social Media Detox and Youth Mental Health. *JAMA Netw Open*. Published November 24, 2025. doi:10.1001/jamanetworkopen.2025.45245

Data
Data available: No
